# Simultaneous experimental evaluation of pulse shape and deadtime phenomenon of GM detector

**DOI:** 10.1038/s41598-021-81571-3

**Published:** 2021-02-08

**Authors:** Bader Almutairi, Syed Alam, Cameron S. Goodwin, Shoaib Usman, Tayfun Akyurek

**Affiliations:** 1 Mining and Nuclear Engineering, Missouri University of Science & Technology, Rolla, MO 65401 USA; 2 Environment and Life Sciences Research Center, Kuwait Institute for Scientific Research, 13109 Kuwait City, Kuwait; 3 Rhode Island Atomic Energy Commission, 16 Reactor Rd, Narragansett, RI 02882 USA; 4 Department of Physics, Faculty of Art and Science, Marmara University, Kadikoy, 34722 Istanbul, Turkey

**Keywords:** Engineering, Nuclear physics

## Abstract

Analysis of several pulse shape properties generated by a Geiger Mueller (GM) detector and its dependence on applied voltage was performed. The two-source method was utilized to measure deadtime while simultaneously capturing pulse shape parameters on an oscilloscope. A wide range of operating voltages (600–1200 V) beyond the recommended operating voltage of 900 V was investigated using three radioactive sources (^204^Tl, ^137^Cs, ^22^Na). This study investigates the relationship between operating voltage, pulse shape properties, and deadtime of the detector. Based on the data, it is found that deadtime decreases with increasing voltage from 600 to 650 V. At these low voltages (600–650 V), the collection time was long, allowing sufficient time for some recombination to take place. Increasing the voltage in this range decreased the collection time, and hence deadtime decreased. It is also observed that rise and fall time were at their highest at these applied voltages. Increasing the voltage further would result in gas multiplication, where deadtime and pulse width are observed to be increasing. After reaching the maximum point of deadtime (~ 250 µs at ~ 700 V), deadtime started to exponentially decrease until a plateau was reached. In this region, it is observed that detector deadtime and operating voltage show a strong correlation with positive pulse width, rise and fall time, cycle mean, and area. Therefore, this study confirms a correlation between detector deadtime, operating voltage, and pulse shape properties. The results will validate our hypothesis that deadtime phenomena at different operating voltages are phenomenologically different.

## Introduction

Researchers have been using Geiger Mueller (GM) counter for almost a century^1^. To detect and record two independent radiation events, there has to be a minimum time interval between two radiation events. In this short interval time, the detector is unresponsive (dead). Any radiation event that takes place within this short interval time will be lost (uncounted). Several studies have shown that the GM counter suffers from a large deadtime compared to other radiation detectors such as solid-state detectors and scintillators^2–4^. The large deadtime that the GM counter suffers from can be from a few microseconds to more than a few milliseconds^5,6^. Moreover, the deadtime phenomenon in radiation detectors has been studied as early as the 1940s. Research on the deadtime phenomenon since then has recognized several factors that affect deadtime, such as the detector’s specifications and design, pulse processing of the detection measurement system, and operating conditions^7^. Mainly, two factors contribute toward the deadtime of a radiation detection system: (I) the inherent deadtime of the detector itself known as intrinsic deadtime, and (II) the collective deadtime that results from pulse processing instruments^8^.

The pulse processing electronics of a typical radiation detection system include a detector, preamplifier, amplifier, discriminator, counter, and multi-channel analyzer (MCA). Nonetheless, for the GM detector, the contribution factor of deadtime from the pulse processing electronics is negligible compared with the detector’s processes. Hence, the intrinsic deadtime is the major contributor to the final deadtime for any GM counting system. Therefore, intrinsic deadtime is sufficient for count rate correction for the case of GM counter.

Due to the fact that radiation events are random, many radiation events go undetected due to the deadtime phenomenon. Subsequently, several deadtime models have been proposed for count rate corrections of the radiation detection system.

### Deadtime models

For count rate correction consideration, there are two traditional deadtime models: (1) the paralyzing, and (2) non-paralyzing models. These two idealized models are employed extensively in the industry and academia. Both models were derived by Feller^9^ and Evans^10^. The paralyzing model assumes that each radiation event taking place within the detector would extend the resolving time (deadtime). If a subsequent radiation event occurs within the extended time, it will not be detected—the count is lost. For that reason, the paralyzing model is known as an extending type. For a radiation event to be counted, there has to be a minimum gap time between two radiation events so that the continuous paralysis of the detector system has lapsed. The proposed paralyzing model, henceforth, attempts to correct the measured count rates. The mathematical description of the paralyzing model is seen in Eq. (1).

1 $$m=n\, {e}^{-n\tau }$$

where $n$ is the true count rate, $m$ is the measured count rate, $\tau$ is deadtime. In contrast, the non-paralyzing model (known as non-extending type) is based on the assumption that each radiation event taking place within the detector will be followed by deadtime. However, when a subsequent interaction takes place during this deadtime, there is no extension of deadtime. Unlike the paralyzing model’s assumption of continuous paralysis of the detector when a radiation event is detected, the non-paralyzing model assumes that the detector is dead only for a fixed time following the detection of a radiation event. The mathematical expression for the non-paralyzing model is given in Eq. (2).

2 $$ m = \frac{n}{{\left( {1 + n\,\tau } \right)}} $$

Further investigation of the deadtime phenomenon in 1978 resulted in a generalized deadtime model derived by Muller^11,12^. By combining the fundamentals of the idealized models, a hybrid deadtime model was later developed by Albert and Nelson^13^, which, in turn, was enhanced by Lee and Gardner^14^. They used Manganese (^56^Mn) radioactive source method for measuring deadtime. Lee and Gardner applied the least fitting square method on the data generated from their experiment. The mathematical expression of the hybrid model is given in Eq. (3). In an effort to improve the hybrid model further, Patil and Usman^6^ proposed another modification by introducing a probability-based paralysis factor ($f$). The paralysis factor was proposed to be between 0 and unity. Equation (4) shows the mathematical expression of their modified hybrid model.

3 $$ m = \frac{{n\,e^{{ - n _{P} }} }}{{1 + n _{NP} }} $$

4 $$ m = \frac{{n\,e^{ - n f } }}{{1 + n \, \tau \left( {1 - f} \right)}} $$

where $\tau_{p}$ is the paralyzing deadtime, *τ*_*NP*_ is the non-paralyzing deadtime, τ is the total deadtime, and $f$ is the probability-based paralysis factor. It is worth noting that if the $f$ is set to 0, the modified hybrid model reduces to the non-paralyzing model, while if $f$ is set to 1, Eq. (4) reduces to the paralyzing model. Additionally, if the $f$ is set to 0.5, deadtime behavior will be lying between the paralyzing and non-paralyzing models. In order to estimate $f$, Patil and Usman proposed a graphical technique. Details of the graphical technique are beyond the scope of this paper and can be found elsewhere^6^. The focus of this manuscript is to understand the phenomenological basis of gas filled detector’s deadtime. As recently reported that using a single deadtime for all possible operating voltages is inappropriate^7^. Purpose of this study is to identify the need of independent deadtime model for various operating voltage range.

### Deadtime behavior

**Figure 1:**
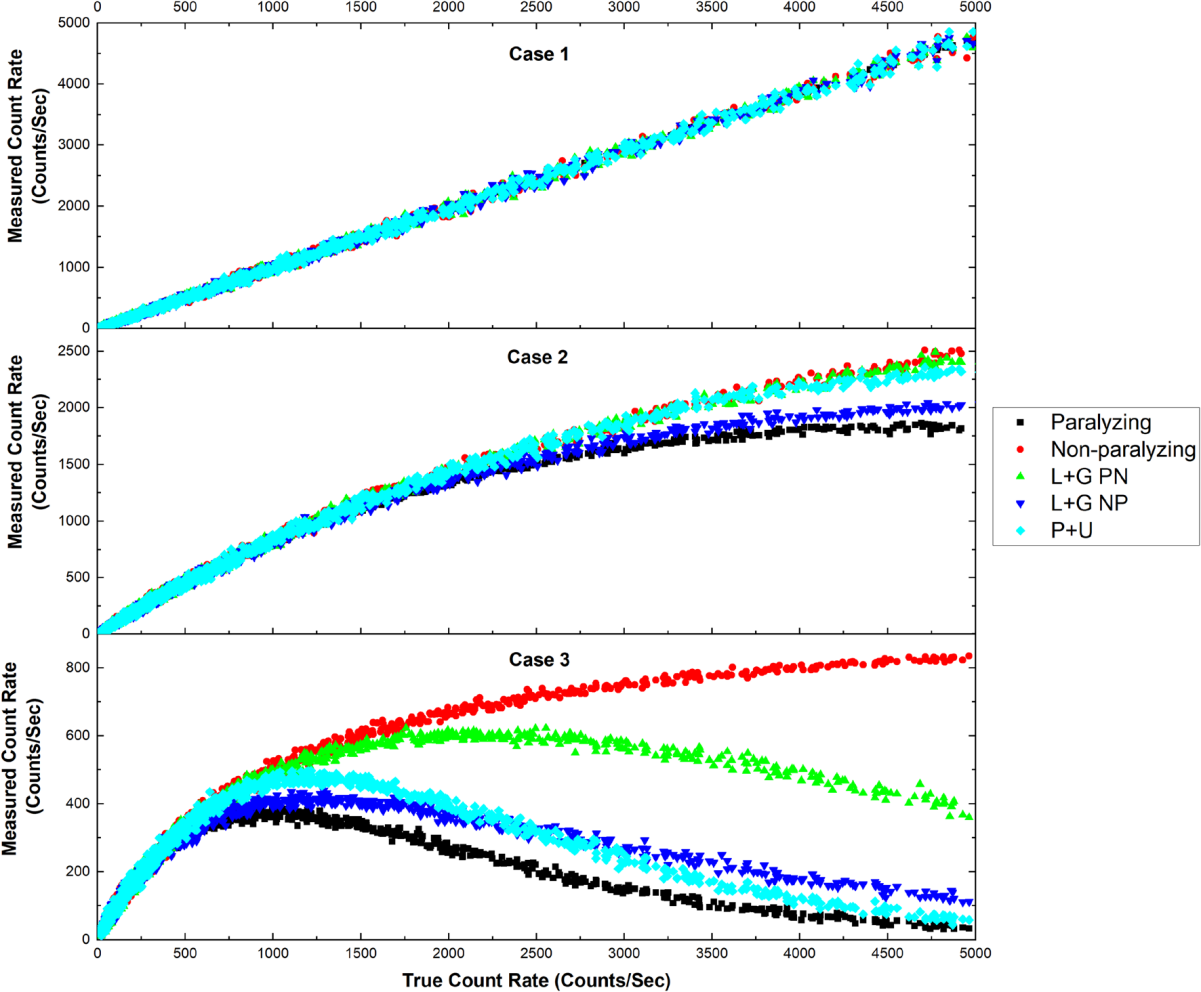
Different deadtime behaviors according to five models. **(**OriginPro 2020b, https://www.originlab.com/2020bAnnouncement).

It was pointed out that the two ideal deadtime models (paralyzing and non-paralyzing) are mathematical convenience rather than phenomenologically based^6,15^. As discussed earlier^8^, the non-paralyzing model uses the first term(s) of Taylor’s expansion of the paralyzing model. Yousaf et al.^15^ developed a simulation code “Sim-Pulse V1.1” to test various deadtime models, ideal and hybrid. We programmed an updated version in MATLAB (R2018b, www.mathworks.com/products/new_products/release2018b), “Sim-Pulse V1.2”, to simulate a short decaying radioactive source (^137m^Ba, with a half-life of 153.12 s) to illustrate the various deadtime behaviors according to the deadtime models discussed in “Deadtime models”. ^137m^Ba is widely utilized for half-life measurement experiments. Three cases were simulated to demonstrate that the choice of model is significant only when the true count rate is high for a GM counter (~ 1000 counts/s, as shown in Fig. 1 for case 3), and the deadtime is long, as shown in Table 1 for case 3. An initial count rate of 5000 counts per second was used for the three proposed cases.

**Table 1:** Total deadtimes used for simulations for each of the 3 cases.

Model	P	NP	Lee & Gardner (NP, PN)	Patil & Usman (NP, PN) (${\varvec{f}}$ = 50%)
**Case 1**				
Total deadtime (µs)	10	10	5 + 5	50%
**Case 2**				
Total deadtime (µs)	200	200	100 + 100	50%
**Case 3**				
Total deadtime (µs)	1000	1000	500 + 500	50%

P stands for the Paralyzing model and NP for the non-paralyzing model. In Lee & Gardner’s model, NP stands for the non-paralyzing model followed by paralyzing, whereas PN is the opposite.

In order to collect the counts for performing the simulations, a bin size of 1 s was used to collect the counts. For GM counters where deadtime is considerably longer than other detectors, the choice of model is significant even at low count rates. However, the general consensus is that the GM detector behaves like an ideal non-paralyzing detector^14^. The behavior of other types of detectors must be carefully evaluated at high count rates before applying any count rate correction.

From Fig. 1, it can be seen that when deadtime is low (10 µs, as shown in Table 1 for case 1), there is little loss of counts, the true count rate after applying deadtime corrections approximately similar to the measured count rate. The choice of the deadtime model for count rate corrections has no serious consequence. Therefore, all deadtime models behave similarly when the deadtime of the detector is low.

In contrast, when deadtime is higher (200 µs and 1000 µs, as shown in Table. 1), all models diverge (as shown in Fig. 1 for cases 2 and 3). In both proposed cases, the paralyzing and non-paralyzing models always set the lowest and highest limits for the true count rate correction, respectively.

It is worth addressing that these traditional models have been applied commonly in radiation detection measurements^16^. Previous studies^6,17,18^ have shown that true deadtime behavior falls somewhere between the idealized models.

### Pulse shape characteristics

In the radiation measurement community, it is widely believed that the pulses produced in a GM detector carry no useful information since the generated pulses have the same amplitude^4,16^. However, this belief has been questioned by recent studies where pulse shape properties were investigated with varying applied voltages^7,19^. It is worth noting that Akyurek et al.^7^ investigation of the pulse shape was performed to confirm the hypothesis that at low voltages, deadtime decreases with increasing voltages until a plateau is reached, and after that, at higher voltages, deadtime increases. Their study focused on pulse duration (pulse width) and the time interval between two pulses (gap time). The investigated operating voltages were in the range of 800 V. It was revealed that at low voltages, pulse width decreases with increasing operating voltage. This reduction of pulse width was attributed to smaller charge collection and hence reduced deadtime. Moreover, at higher operating voltages, the second pulse after a long width pulse was observed to be of short duration. This reduction in the second pulse was attributed to smaller charge production for the second event during the recovery time. Although the initial work by Akyurek et al.^7^ was very interesting, it lacked the analysis of several other important pulse shape properties such as amplitude, fall time, rise time, area, and positive pulse width. It is also worth noting that the generated pulses in their study were manually captured using an oscilloscope. The use of automatic measurements offered by an advanced oscilloscope would have shown more details on pulse shape properties.

In an effort to study the generated pulse properties from a GM counter even further, we designed an experiment that used two different radioactive sources (^60^Co and ^137^Cs). The details of the study were discussed and can be found in a recently published study^19^. The recommended operating voltage specified by the manufacturer for that particular GM detector (Ludlum, model 133-2) was 550 V. A wide range of voltages from 300 to 1000 V was examined in the previous study. Nonetheless, we did not examine the pulses at voltages above 1000 V because it would damage the detector. The study concluded that the detected pulses from both radioactive sources behaved similarly in which pulse width and fall time were exponentially decreasing with increasing the operating voltages. In contrast, peak-to-peak (Pk to Pk) increased with increasing voltages until an asymptote was observed at the highest operating voltages. Pulse shape dependence on operating voltage for a GM counter was discussed in detail, but simultaneous deadtime and pulse shape measurements were missing. Therefore, no relationship between deadtime and pulse shape could be deduced from the earlier work^19^.

The purpose of this work is to examine pulse shape properties and its relationship with observed deadtime more in-depth. An experiment was designed where deadtime and generated pulses were simultaneously measured and recorded. Four different radioactive sources were used: ^204^Tl, ^137^Cs, ^22^Na, and ^54^Mn. Measurements of deadtime and its associated behavior were discussed in our previous research paper^20^. Based on the findings, the phenomenological basis of deadtime manifestation was presented. Three distinct ranges of deadtime phenomenon depending on the operating voltage were identified, and for each range, a phenomenological model was presented. For the GM detector (Ludlum, model 44–7) tested in the study, these regions were: (I) region 1 (600–650 V), (II) region 2 (700–750 V), and (III) region 3 (750–1200 V).

Limited literature is available about deadtime dependence on applied voltages, but not much discussion is available about the relationship between pulse shape generated in a GM counter and detector deadtime. Therefore, this study appears to be the first attempt to present information on the correlation between GM deadtime and pulse shape, which would help the radiation measurement community better understand the deadtime phenomenon. The results will validate our hypothesis that deadtime phenomena at different operating voltages are phenomenologically different.

## Experimental methods

### Experimental details

**Figure 2:**
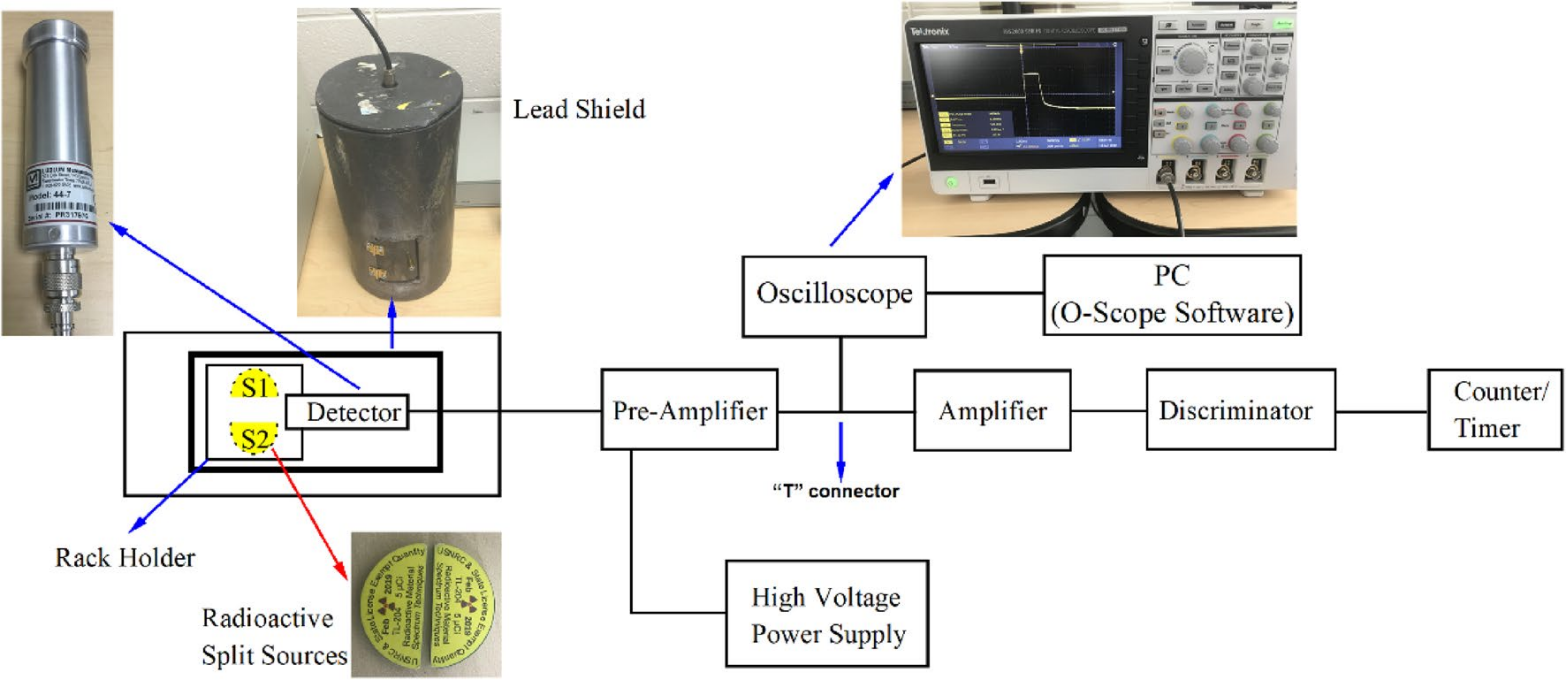
A schematic of the experimental setup for the radiation counting system.

Figure 2 shows a schematic of the experimental setup used to measure the deadtime of the GM detector and record the output train of pulses due to radiation interactions. The counting system in this experiment consisted of radioactive half-disk sources, GM detector, high-voltage power supply, preamplifier, oscilloscope, amplifier, integral discriminator, dual counter/timer, and a PC.

The radioactive sources used in this experiment were (a) ^204^Tl, (b) ^137^Cs, and (c) ^22^Na. These sources were designed and produced specifically by Spectrum Techniques LLC. upon our request to conduct this study. ^204^Tl was produced in February 2019, while the other two radioactive sources were produced in May 2019. Each source consists of two sealed half-disk sources, as can be seen in Fig. 2. From now and onward, we will refer to the half-disk source as a split source. The initial activity of each split source was 5 µCi, with ± 20% uncertainties^21^. Due to the fact that the radioactive sources were not short-lived isotopes (seconds, minutes, hours) no correction to the deadtime due to decay during measurement was needed^22^. To ensure that the uncertainties associated with each split source do not result in significant statistical errors, we conducted several evaluations of each split source of the three isotopes. The outcomes showed that each split source resulted in a similar number of observed counts. Hence, the uncertainties did not inflict significant statistical errors. In addition, we used the same size and geometry to minimize errors from measuring the radioactive sources.

The GM detector used in this study was a halogen-quenched, end-window type detector with a 4π geometry. The dimension of the GM detector (height and diameter) is 14.7 cm × 4.6 cm. The end window was made out of a thin mica layer to detect not only gamma radiation but also alpha and beta rays. The recommended operating voltage of this GM detector was 900 V, while the typical deadtime associated with this model was 200 µs, as specified by the manufacturer^23^.

The low-noise charge-sensitive preamplifier (Ortec, model 142A) was connected through “E” input with a connector series “C” with a short coaxial cable to the GM detector^24^. To minimize noise and maintain the preamplifier’s stability, it was placed as close as possible to the detector. The preamplifier’s capacitor feedback was 1 pF while the pulse tail decays to the baseline in 500 µs. In addition, the preamplifier’s bias input was connected to the high-voltage (HV) power supply. Also, the preamplifier was taped to the same location for the subsequent experiments.

The HV power supply (Canberra, model 3125)^25^ was housed in the nuclear instrumentation module (NIM), and it was directly connected to the AC line. The oscilloscope (Tektronix, model TBS2000) was connected to the preamplifier through a “T” connector to capture and record the generated pulses directly from the preamplifier^26^. The purpose was to capture and record the pulse shape properties generated from the GM detector without going through the other pulse processing electronics (amplifier, integral discriminator, dual counter/timer). The oscilloscope’s output was connected to a PC where an O-scope utility software (provided by Tektronix.inc, version 1.5, https://www.tek.com/oscilloscope-software) was used to record measurements of the pulse shape properties automatically. The amplifier (Ortec, model 570) was connected to the preamplifier through a “T” connector using a coaxial cable^27^. Therefore, the preamplifier was connected to both the oscilloscope and the amplifier through the “T” connector (a splitter). The amplifier serves two purposes in general: (1) to amplify the pulse coming from the preamplifier, and (2) to shape the pulse and eliminate the long exponential tail of the pulse processed by the preamplifier so that pile-up of pulses are reduced. Besides, the amplifier was connected to the integral discriminator (Canberra, model 832)^28^. The discriminator was used to produce logic pulses when the linear input pulse’s amplitude processed by the amplifier exceeds a threshold. The discriminator was connected directly to the dual counter/timer (Ortec, model 994)^29^. The dual counter/timer was used to measure the number of registered counts. The timer was set to 30 min for each experiment, and it was used to start and end registering counts from radiation events automatically.

### Two source method

In this current study, we utilized the two-source method to measure deadtime dependence on operating voltages. The principle behind this method is that when two radioactive sources are combined, they will result in fewer observed count rates than if each radioactive source measured individually and summed up. The loss of counts from observing the combined radioactive sources is attributed to deadtime. In our study, we used two split sources for each radioactive isotope. Split source one is referred to as S_1_, while split source two is S_2_. Combined, the split sources are abbreviated as S_12_. Since the GM detector is commonly known to behave as non-paralyzable, and it suffers from ≤ 5% of the paralysis factor, applying the non-paralyzable model for calculating deadtime-voltage dependence in our study is, therefore, justified^6,8,14^. The derivation of the two-source method is well documented in Knoll’s textbook^4^. Equations (5–7) were used to calculate the final deadtime, as given in Eq. (8) of the counting system used in this study.

5 $$ X = s_{1} s_{2} - BKGs_{12} $$

6 $$ Y = s_{1} s_{2} . \left( {s_{12} + BKG} \right) - BKG . s_{12} . \left( {s_{1} + s_{2} } \right) $$

7 $$ Z = \frac{{Y\left( {s_{1} + s_{2} - s_{12} - BKG} \right)}}{{X^{2} }} $$

8 $$ \tau = \frac{{X\left( {1 - \sqrt {1 - Z} } \right)}}{Y} $$

where $s_{1}$, $s_{2}$, $s_{12}$ are defined previously, BKG is background count rate, and $\tau$ is deadtime of the counting system.

Careful measurements were conducted to observe the difference between the large numbers of radiation events detected from $s_{1}$ and $s_{2}$ individually. $s_{1}$ was placed on a marked paper on a tray on the second shelf of the rack holder in a cylindrical lead shield that contains the GM counter, as illustrated in Fig. 2. The same technique was applied for $s_{2}$. The marked paper was used to verify that the split sources of all radioactive isotopes used in this study had the same position. This step was performed in order to ensure that each experiment had the same geometry and location; hence, the same solid angle applied for all experiments. The same technique was utilized for counting the radiation events from $s_{12}$. To achieve optimal results for final deadtime calculation, from choosing the shelf level to carry the split sources to adjusting the processing instrumentations, a fractional deadtime $\left( {s_{12} \tau } \right)$ of at least 20% was acquired.

The duration of each experiment using $s_{1}$, $s_{2}$, and $s_{12}$ was 30 min. Since this current study investigates pulse shape characteristics associated with deadtime behavior, a wide range of operating voltages from 600 to 1200 V with increments of 50 V were examined. The GM counter started registering radiation interactions at 570 V. However, negative deadtimes were obtained at this low operating voltage; consequently, data collection started at 600 V, where positive deadtimes were achieved.

Since this study focused on pulse shape analysis, a detailed description of instrumentation optimizations and deadtime measurements are outlined in an earlier paper^20^.

### Oscilloscope

The oscilloscope (Tektronix, model TBS2000)^26^ was used in this experiment to display and record the train of pulses generated by the GM detector. The oscilloscope’s channel one input was connected to the preamplifier through a “T” connector with a coaxial cable. In contrast, the output was connected directly to the PC to process the data in real-time. Table 2 shows the definition for each pulse property collected and analyzed in this study. The bandwidth of the oscilloscope was in full mode. The oscilloscope’s probe type was voltage, while the probe’s attenuation factor was 10X. The record length for the acquiring option was 20 M points, while the sample rate was 500 MS/s. The Horizontal Scale (time per major horizontal division) was set to 200 µs/div while the Vertical Scale was 50 V/div. The value of the termination resistance was 10 MΩ. To automatically record waveform data, we used the triggering mechanism in which the rising edge trigger condition was selected. The trigger delay mode time was set to negative values in order to capture more waveform data. The trigger source was set to channel 1 with a 20 V slope. The input signal coupling method for the oscilloscope was (DC), which means it passes both the AC and DC signal components. Besides, it passes the trigger signal without filtering it to the trigger circuit.

**Table 2:** Pulse shape measurements with their definitions. These definitions derived from Tektronix’s manual for the TBS2000 oscilloscope.

Pulse property	Definition
Amplitude	It is a measurement over the entire waveform in which it is the average high value of the pulse less the average low value. Its unit is volts
Area	The area over the entire waveform and measured in volts-seconds. It is positive for measurements above ground and negative below the ground
Cycle mean	The arithmetic mean over the first cycle
Fall time	Time measurement in seconds that is measured from the high reference value (90%) to the low reference value (10%) of the final value of the pulse. This is known as the tail of the pulse with an exponential decay
Frequency	The first cycle in a waveform or gated region, measured in Hertz (Hz)
Positive pulse width	The distance (time) between the mid reference (50%) point of a positive pulse
Positive duty	This is a calculated measurement and not measured directly. It is the ratio of the positive pulse width to the signal period in percent
Rise time	Time measurement where it starts from the low reference value (10%) of the leading edge of the pulse to the high reference value (90%) of the final value. For saturated pulses since the oscilloscope is unable to see the full pulse, 10% to 90% rise time is for the truncated pulse
Peak to peak	It is the measurement of absolute difference between the minimum and maximum amplitude in the entire waveform

Furthermore, the data collection duration for each radioactive split source (S_1_ and S_2_) was 30 min, and 30 min for combined sources (S_12_). Data logging by the O-scope software was set to 15 s, which means a total of 120 data points for each pulse property were recorded. Hence, each pulse property’s averages at the specified operating voltage for each experiment were calculated and analyzed. In the subsequent section, we discuss this behavior and its association with deadtime.

## Results and discussion

### Pulse shape analysis

In an earlier study^20^, a two-source method was used to calculate GM counter deadtime for a wide range of operating voltages. Results showed an interesting relationship between the operating voltage and the detector deadtime. At 650 V, deadtime reached a minimum value. From 700 to 750 V, deadtime started to increase rapidly to reach a maximum. After 750 V, an exponential decrease in the deadtime was observed, leading to an asymptotic. This behavior of deadtime variation with operating voltage dependence was consistently using the three radiation sources.

Based on these results, three distinct regions of the GM counter deadtime phenomenon were observed. From 600 to 650 V, the applied voltage was not sufficient to rapidly collect all charges initially produced by the ionization of the gas. Therefore, significant recombination of electrons and positive ions took place before charge collection; hence, not all radiation events are recorded. Looking at the extreme case of zero bias voltage, the charge produced from radiation's initial interaction will recombine. No pulse will be recorded, irrespective of the initial interaction location. As we increase the bias voltage, some initial interaction occurring at high field intensity locations within the chamber will be recorded, while other initial interactions occurring in corners of the gas volume will not be recorded even to the oscilloscope. These are the interactions taking place in locations of weak field intensity where recombination dominates. As we increase the operating voltage, the detector's entire volume experience sufficient voltage; hence, all events are recorded (no recombination), but now the deadtime starts to act, and the loss of count at higher voltages are not due to recombination but rather convolution of pulses. Since there is no method for an experiment to separate the loss of count based on the phenomenon of the loss, recombination-based loss of count is also seen as deadtime losses. Therefore, at low voltages between 600–650 V, deadtime seems to be decreasing. Strictly speaking, this is not because of the deadtime decreasing rather a suppression of recombination.

From 650 to 750 V, the operating voltage reached a point where the velocity of the ions and the electrons were high enough for charge multiplication. Therefore, a larger number of charge carriers were available. Since more charge has to be collected, a rapid increase in deadtime was observed in this intermediate operating voltage range. Because increasing voltage in this range increases charge multiplication, deadtime continued to increase with increasing the voltage until 750 V.

After 750 V, deadtime started to decrease exponentially. The reason behind this exponential decrease in deadtime with increasing operating voltages was because no additional charge multiplication was possible. Hence, the collection time was reduced. The recommended operating voltage (900 V) of the GM counter was at the low asymptotic value of deadtime.

Previous studies attempted to obtain a relationship between applied high voltage and deadtime^7,19^, but the relationship between pulse shape properties and deadtime for GM detector has not been investigated. Therefore, a comprehensive experimental campaign was designed and executed to collect data on deadtime and the various parameters describing the pulse shape. For this study, we only focused on the high voltage/GM region, which is of primary interest to many applications. For this study, ^204^Tl, ^137^Cs, ^22^Na sources were used to examine the relationship between applied high voltage, GM counter deadtime, and pulse shape properties.

**Figure 3:**
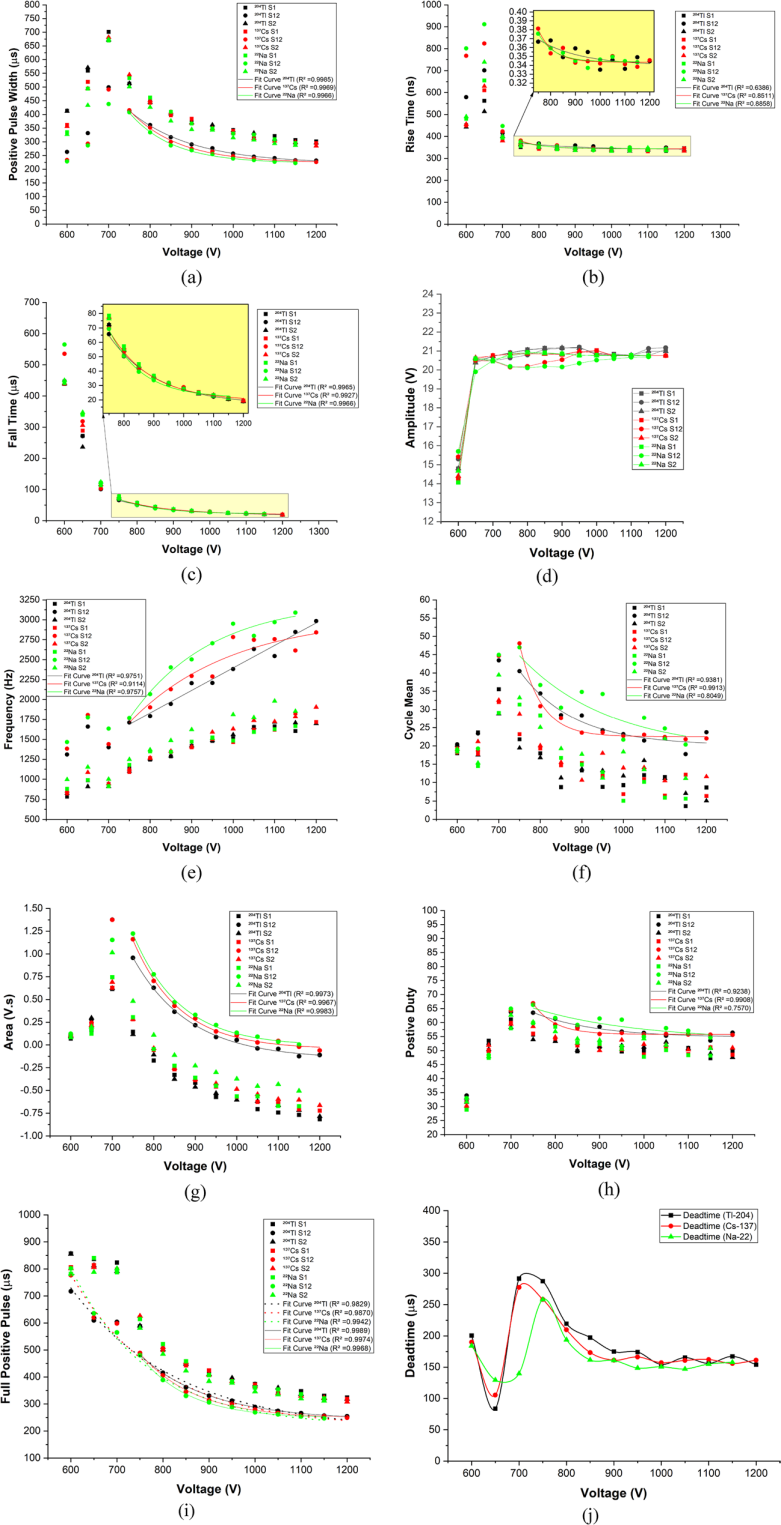
Different parameters with respect to applied high voltage for pulse shape analysis. (OriginPro 2020b, https://www.originlab.com/2020bAnnouncement).

Figure 3a shows positive pulse width with respect to applied voltages. At 600 V, it is seen that the pulse width is at its shortest. As discussed above, recombination was significant in this region; hence, the charge collection was incomplete, and not many pulses were detected. At 700 V, the ionization process took place; thus, the generated pulse was larger. From 700 to 750 V, the gas multiplication process took over and resulted in the widest pulse width. After 750 V, gas multiplications produced Townsend avalanches; hence, the detected pulses started to shrink in width. Furthermore, it can be seen from Eq. (9) that the duty cycle depends on the pulse width and the period; henceforth, there is a direct relationship between the period of the pulse and pulse width. Figure 3a,h show that the pulse width decreased while the duty cycle was almost constant after 700 V.

9 $$ {\text{Duty}} \;{\text{Cycle}} = {\text{Pulse}} \;{\text{width}}/{\text{Period}} $$

From Fig. 3b, it can be seen that the rise time between 600 to 650 V was relatively high. This is because recombination processes at these low voltages were prominent; hence, many radiation events were not detected. At the same time, the charge collection time was long, leading to a long rise time. From 700 V and onward, the voltage increase combined with the ionization processes, resulted in faster collection time. Therefore, it is observed in this region that rise time was exponentially decreasing with increasing operating voltage.

Next, it can be seen from Fig. 3c that fall time was at its highest at 600 V. This means that the pulse had a longer tail because the applied voltage was not strong enough. Therefore, the charge collection time was longer, which agrees with the rise time observation. As the voltage was increased further, it is observed that fall time was exponentially decreasing because more radiation events were detected.

Moreover, it can be seen from Fig. 3d that amplitude was at its lowest at 600 V. The reason behind this is that recombination processes did not produce a full charge (weak current). Nonetheless, more pulses were detected as voltages increased further. The Townsend avalanches were continuously produced until a sheath of positive ions was formed around the anode, decreasing the electric field below the point where additional gas multiplication cannot occur. The process finished when the GM counter produced the same total number of positive ions b created by the incident radiation. At this point, the GM counter generates the same amplitude for each output pulse. This outcome can be precisely observed after 700 V, as shown in Fig. 3d.

Figure 3e shows the frequency of the pulses as a function of applied voltages. The shape of frequency behavior was similar to the shape of the observed radiation counts. For the region of interest, 750 V and above, it is seen that as the frequency was increased, more counts were detected. The higher the voltage, the faster a pulse was detected, while the shorter distance between pulses was observed.

Furthermore, the definition of cycle mean is the arithmetic mean over the first cycle in the waveform or the first cycle in the gated region. The cycle mean is a part of the amplitude measurement category, as explained in the oscilloscope manual. Figure 3f shows the cycle mean as a function of operating voltages. The cycle mean behavior was similar to the deadtime behavior of the GM detector, where deadtime tends to exponentially decrease after 750 V. The area of a pulse can be calculated as amplitude times pulse width. It is observed from Fig. 3g that area is decreasing exponentially after 750 V. This is expected because the amplitude was constant while the pulse width was exponentially decreasing after 750 V. Looking at the figures of “Area” and “Cycle mean” with respect to the applied high voltages, it is observed that they follow a similar behavior of deadtime.

Positive duty is defined in signals as pulse width divided by the period times 100%. It is a calculated value not directly measured. For instance, it is seen from Fig. 3h that at 600 V, the positive duty cycle was approximately 30%, which means that the pulse width occupies 30% of the period or the signal is ‘on’ 30%. Hence, the pulse width was short at this voltage, as observed in Fig. 3a. At 650 V, positive duty was 50%, which means that the pulse width occupied half of the period. From 750 V and onward, positive duty for combined radioactive sources was above 55%, which means that the signal was ‘on’ 55% of the time, and it was exponentially decreasing with increasing voltages.

Figure 3i shows the full size of the positive pulse (rise time + pulse width + fall time). When all of these properties were added together, it is observed that from 600 to 1200 V, the full positive pulse was exponentially decreasing. This behavior is also in agreement with the findings of our previous study^19^. Table 3 summarizes the statistics of the fit lines in Fig. 3. Since this study focuses on higher applied voltages and the GM region, the exponential fittings were performed from 750 to 1200 V. The error bars are not included in the figures because the software used to collect pulse characteristics automatically did not provide any statistical error. Deadtime behavior of the three radioactive isotopes as a function of applied voltage is shown in Fig. 3j. Deadtime was manually calculated together with the error but the error bars are too small to be visible in Fig. 3.

**Table 3:** Statistical parameters for best fit lines in Fig. 3.

**Pulse property**	**Positive pulse width**		
Equation	$y = y_{0} + A*e^{{\left( {R_{0} *x} \right)}}$		
Source	^204^Tl S12	^137^Cs S12	^22^Na S12
*Y*_0_	2.20E−4 ± 2.79E−6	2.23E−4 ± 2.98E−6	2.21E−4 ± 3.2E−6
*A*	0.0270 ± 0.00577	0.110 ± 0.0395	0.239 ± 0.109
*R*_*0*_	− 0.0066 ± 2.91E−4	− 0.0084 ± 4.78E−4	− 0.0095 ± 6.1E−4
R-square	0.99851	0.99687	0.99657
**Pulse property**	**Rise time**		
Equation	$y = y_{0} + A*e^{{\left( {R_{0} *x} \right)}}$		
Source	^204^Tl S12	^137^Cs S12	^22^Na S12
*Y*_0_	3.39E−6 ± 9.83E−8	3.43E−6 ± 2.51E−8	3.43E−6 ± 2.3E−8
*A*	2.33E−5 ± 9.14E−5	0.094 ± 0.44	0.033 ± 0.14
*R*_*0*_	− 0.0058 ± 0.0054	− 0.016 ± 0.0062	− 0.015 ± 0.0054
R-square	0.6386	0.8511	0.8858
**Pulse property**	**Fall time**		
Equation	$y = y_{0} + A*e^{{\left( {R_{0} *x} \right)}}$		
Source	^204^Tl S12	^137^Cs S12	^22^Na S12
*Y*_0_	1.68E−5 ± 1.16E−6	1.91E−5 ± 1.39E−6	2.15E−5 ± 8.6E−7
*A*	0.00498 ± 0.00158	0.0117 ± 0.00586	0.0455 ± 0.0200
*R*_*0*_	− 0.0062 ± 4.37E−4	− 0.0073 ± 6.76E−4	− 0.0091 ± 5.9E−4
R-square	0.99649	0.99273	0.99664
**Pulse property**	**Frequency**		
Equation	$y = y_{0} + A*e^{{\left( {R_{0} *x} \right)}}$		
Source	^204^Tl S12	^137^Cs S12	^22^Na S12
*Y*_0_	105,526.02 ± 3,603,694.81	3043.73 ± 310.95	3232.99 ± 157.15
*A*	− 105,991.02 ± 3,602,417.9	− 27,797.84 ± 38,118.4	− 69,897.5 ± 62,645
*R*_*0*_	− 2.73E−5 ± 9.55E−4	− 0.0040 ± 0.0020	− 0.0051 ± 0.0012
R-square	0.97506	0.91143	0.9757
**Pulse property**	**Cycle mean**		
Equation	$y = y_{0} + A*e^{{\left( {R_{0} *x} \right)}}$		
Source	^204^Tl S12	^137^Cs S12	^22^Na S12
*Y*_0_	20.13 ± 1.63	22.51 ± 0.36	18.51 ± 10.20
*A*	6460.25 ± 9949.45	5.96E7 ± 7.55E7	835.30 ± 2228.27
*R*_*0*_	− 0.0076 ± 0.0021	− 0.01956 ± 0.00168	− 0.0046 ± 0.0039
R-square	0.9381	0.99131	0.80486
**Pulse property**	**Area**		
Equation	$y = y_{0} + A*e^{{\left( {R_{0} *x} \right)}}$		
Source	^204^Tl S12	^137^Cs S12	^22^Na S12
*Y*_0_	− 0.154 ± 0.019	− 0.047 ± 0.018	− 0.012 ± 0.017
*A*	274.04 ± 83.42	927.10 ± 351.57	885.89 ± 273.52
*R*_*0*_	− 0.0073 ± 4.12E−4	− 0.0088 ± 5.07E−4	− 0.0087 ± 4.1E−4
R-square	0.9973	0.99666	0.99825
**Pulse property**	**Positive duty**		
Equation	$y = y_{0} + A*e^{{\left( {R_{0} *x} \right)}}$		
Source	^204^Tl S12	^137^Cs S12	^22^Na S12
*Y*_0_	54.66 ± 0.88	55.78 ± 0.15	53.72 ± 5.65
*A*	1771.764 ± 2910.64	6.37E7 ± 8.92E7	294.57 ± 878.59
*R*_*0*_	− 0.0070 ± 0.0022	− 0.021 ± 0.0019	− 0.0043 ± 0.0044
R-Square	0.92379	0.99083	0.75704
**Pulse property**	**Full positive pulse**		
Equation	$y = y_{0} + A*e^{{\left( {R_{0} *x} \right)}}$		
Source	^204^Tl S12	^137^Cs S12	^22^Na S12
*Y*_0_	2.41E−4 ± 3.09E−6	2.461E−4 ± 3.54E−6	2.47E−4 ± 4.0E−6
*A*	0.03197 ± 0.00592	0.1185 ± 0.03808	0.28506 ± 0.125
*R*_*0*_	− 0.007 ± 2.54E−4	− 0.008 ± 4.32E−4	− 0.001 ± 5.89E−4
R-square	0.9989	0.9974	0.9968
**Pulse property**	**Full positive pulse**		
Voltage range	600–1200 V		
Equation	$y = y_{0} + A*e^{{\left( {R_{0} *x} \right)}} \frac{{\partial^{2} \Omega }}{{\partial v^{2} }}$		
Source	^204^Tl S12	^137^Cs S12	^22^Na S12
*Y*_0_	2.017E−4 ± 2.61E−5	2.14E−4 ± 1.88E−5	2.16E−4 ± 1.3E−5
*A*	0.0071 ± 0.0024	0.0129 ± 0.0042	0.0201 ± 0.0051
*R*_*0*_	− 0.004 ± 5.97E−4	− 0.005 ± 5.57E−4	− 0.006 ± 4.31E−4
R-square	0.9829	0.9870	0.9942

OriginPro software (version 2020b) was used to derive best fit lines.

### Exponential fitting models

**Figure 4:**
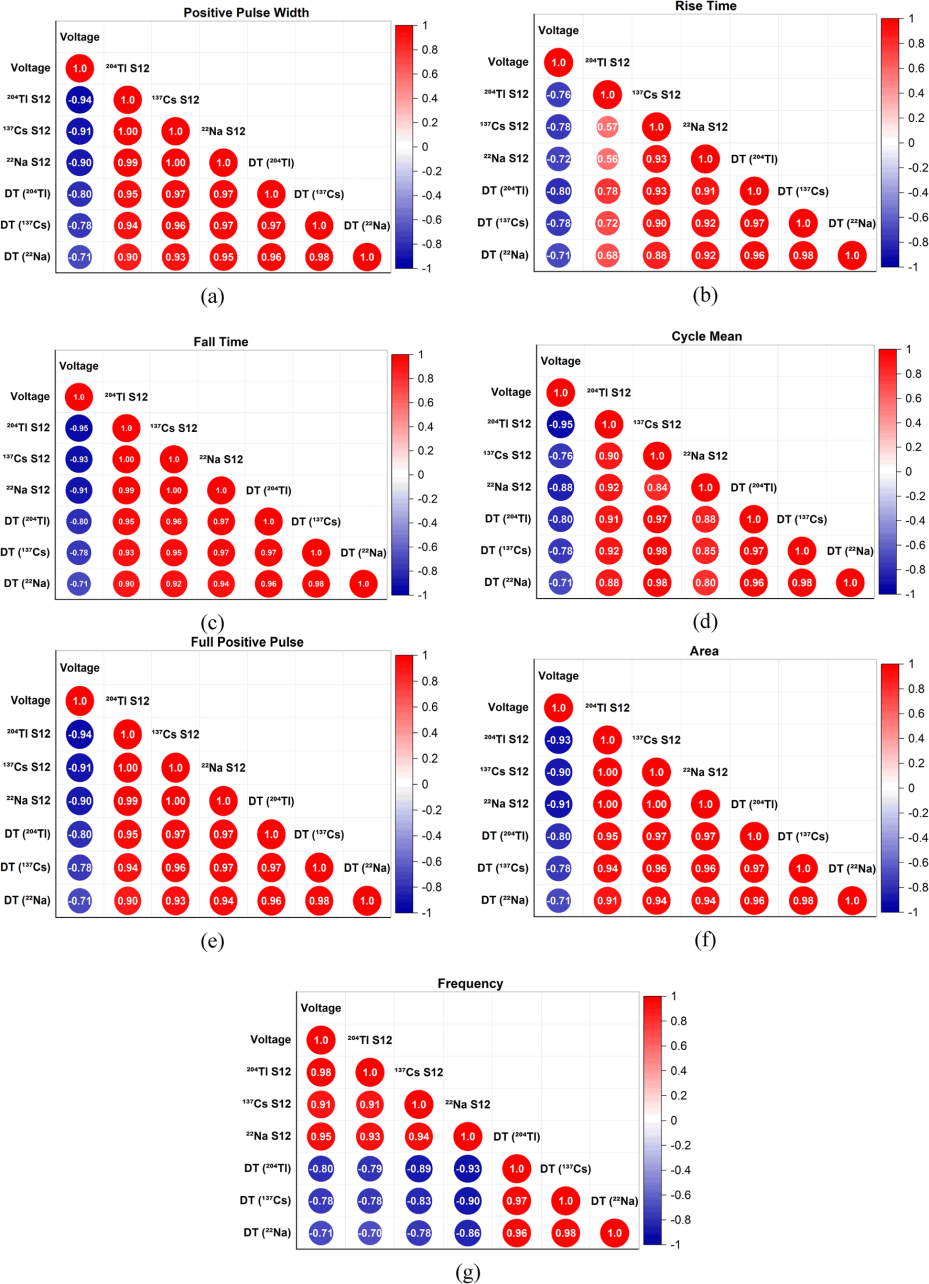
Correlation coefficients for pulse shape characteristics and operating voltages from 750 to 1200 V. S12 stands for combined radioactive sources. DT stands for deadtime.

Figure 4 shows the result of some detailed statistical analysis of the results. Since the GM region is of primary interest to many applications, we focused on high applied voltages (from 750 to 1200 V). Also, since individual radioactive sources showed similar behavior when combined sources were used, we focused our correlations analysis on combined sources. Furthermore, various pulse shape characteristics were examined for their dependence on the operating voltage and its relation to deadtime.

Figure 4a shows a negative correlation between the operating voltage and the “Positive Pulse Width.” For all three radioactive sources (^204^Tl, ^137^Cs, ^22^Na), the value of the coefficient ranges from − 0.94 to − 0.90, which is a very strong correlation. However, for all three sources, a weaker correlation between − 0.80 and − 0.71 was observed with deadtime. Based on the data, one can deduce a strong positive correlation between pulse width and deadtime with coefficients ranging between 0.96 and 0.95, as shown in Fig. 4a. This means that the longer the pulse width, the higher the probability for overlapping pulses; hence, deadtime was at its maximum point (at 750 V) when pulse width was observed to be at its maximum width, as can be seen in Fig. 3a. As the applied voltage increases, the shorter the pulse width, the more counts were measured. After 750 V, the detector started to operate in the GM region; hence, deadtime started to decrease exponentially after 750 V until a plateau was reached, as shown in Fig. 3a.

When observing Fig. 4b, one is bound to notice a strong negative correlation between the operating voltage and the pulse “Rise Time” with coefficients ranging between − 0.78 and − 0.72 for combined sources. Again, there is a positive correlation between deadtime and the pulse “Rise Time.” This means that the smaller the “Rise Time”, the faster was the collection time of charge due to the increased applied voltage; hence, deadtime showed an exponential decrease until a plateau was reached, as shown in Fig. 3b.

Similar results were recorded (Fig. 4c) for the correlation between the operating voltage and the pulse “Fall Time” with the only difference that the correlation was stronger with the operating voltage (− 0.95 to − 0.91). Also, pulse “Fall Time,” shows a strong correlation with deadtime (0.95 to 0.94). Almost identical results were observed for the operating voltage correlation with “Cycle Mean,” “Full Positive Pulse,” and “Area” (Fig. 4d–f).

Nevertheless, the results for the pulse “Frequency” correlation with operating voltage is quite interesting, as shown in Fig. 4g. A strong positive correlation was observed (between + 0.98 to + 0.91) for all sources. This means that increasing the operating voltage increases the count rate. Furthermore, at high frequency, the count rate increased, leading to a higher probability for overlapping pulses; however, because the voltage was higher in which the GM detector operates in the GM region, the deadtime plateau. Pulse “Frequency” and deadtime show a negative correlation between − 0.86 and − 0.79, as shown in Fig. 4g.

## Conclusions

To the best of authors’ knowledge, this is the first attempt to correlate GM counter operating voltage with pulse shape characteristics and detector deadtime. Based on the data collected in this study, one can draw the following conclusions:

- The general belief that for any GM counter pulse amplitude, pulse shape, and deadtime is constant for the entire operating voltage range is incorrect, as recently shown by Akyurek^7^ and Almutairi^19^. From 600 to 650 V, deadtime decreases. From 650 to 750 V, deadtime increases rapidly until a maximum deadtime was reached. After 750 V, deadtime exponentially decreases until a plateau was reached.
- Akyurek et al.^7^ provided some interesting data but their work lacked the analysis of several other important pulse shape properties such as amplitude, fall time, rise time, area, and positive pulse width.
- Data on deadtime and the various pulse parameters were simultaneously collected using ^204^Tl, ^137^Cs, ^22^Na sources to examine the relationship amongst the applied high voltage, GM counter deadtime, and pulse shape properties.
- Based on the data, three distinct deadtime phenomenon depending on the operating voltage were observed.
- At the lowest applied voltages (600 to 650 V), the deadtime was caused by charge recombination. Increasing the voltage increases pulse width; hence, deadtime was reduced.
- It can be seen in Fig. 3c that rise and fall time were at their highest at 600 V. This means that the pulse had a longer tail because the applied voltage was not strong. Therefore, the charge collection time was longer, leading to the long deadtime. Both pulse width and deadtime were reduced with increasing voltage in this region.
- When the voltage was high enough for charge multiplication, the deadtime and pulse positive width started to increase. This was due to the fact that more time was needed to collect a larger number of charge carriers.
- At the end of the proportionality region, no additional multiplication was possible due to the reduced spaced field intensity after reaching the maximum deadtime.
- After the point of maximum deadtime, there was an exponential drop in deadtime in the GM region until a plateau was reached. This plateau value for the detector tested in these experiments was approximately 150 µs. The manufacturer’s Data Sheet provided the value as “typically 200 microseconds.” This difference is because it is difficult to control all the engineering processes and tolerances. Hence, the individual detector could vary in their deadtime values. Value of deadtime also depends on the operating conditions as reported here.
- Operating voltage and detector deadtime exhibit strong correlation with various pulse properties like positive pulse width, rise and fall time, cycle mean, full positive pulse, and area.
- These results are significant for GM counters, indicating the need for a specific voltage-dependent deadtime models. These models will allow a better understanding of deadtime correction and reduce reliance on empirical correlations and calibration-based corrections. This is particularly important for the fission chamber, BF_3,_ and He detectors used for reactor applications.

## References

[1] Geiger H (1908). On the scattering of the α-particles by matter. Proc. R. Soc. Lond. A.

[2] Geiger H, Müller W (1928). Electron counting tube for the measurement of the weakest radioactivities. The Sciences.

[3] Skinner SM (1935). The efficiency of the tube counter. Phys. Rev..

[4] Knoll GF (2010). Radiation Detection and Measurement.

[5] Muller JW (1973). Dead-time problems. Nucl. Instrum. Methods.

[6] Patil A, Usman S (2009). Measurement and application of paralysis factor for improved detector dead-time characterization. Nucl. Technol..

[7] Akyurek T, Yousaf M, Liu X, Usman S (2015). GM counter deadtime dependence on applied voltage, operating, temperature and fatigue. Prog. Nucl. Energy.

[8] Usman S, Patil A (2008). Radiation detector deadtime and pile up: A review of the status of science. Nucl. Eng. Tech..

[9] Feller, W. On probability problems in the theory of counters. In: *R. Courant Anniversary Volume, Studies and Essays* 105–115 (Wiley Interscience, New York, 1948).

[10] Evans RD (1955). The Atomic Nucleus.

[11] Muller, J. W. *A simple derivation of the Takacs formula*. (Bureau International des Poids et Mesures, 1988).

[12] Muller JW (1991). Generalized dead times. Nucl. Instrum. Methods Phys. Res. A..

[13] Albert GE, Nelson L (1953). Contributions to the statistical theory of counter data. Ann. Math. Stat..

[14] Lee SH, Gardner RP (2000). A new G-M counter dead time model. Appl. Radiat. Isot..

[15] Yousaf M, Akyurek T, Usman S (2015). A comparison of traditional and hybrid radiation detector dead-time models and detector behavior. Prog. Nucl. Energy.

[16] Tsoulfanidis N, Landsberger S (2015). Detector dead-time correction and measurement of dead time. Measurement and Detection of Radiation.

[17] Akyurek T, Tucker LP, Liu X, Usman S (2016). Portable spectroscopic fast neutron probe and 3He detector dead-time measurements. Prog. Nucl. Energy.

[18] Costrell L (1949). Accurate determination of the deadtime and recovery characteristics of Geiger-Muller counters. J. Res. Natl. Bur. Stand..

[19] Almutairi B, Akyurek T, Usman S (2019). Voltage dependent pulse shape analysis of Geiger-Müller counter. Nucl. Eng. Tech..

[20] Almutairi B, Alam S, Akyurek T, Goodwin C, Usman S (2020). Experimental evaluation of the deadtime phenomenon for GM detector: deadtime dependence on operating voltages. Sci Rep.

[21] Disk Source Sets. http://www.spectrumtechniques.com/products/sources (2020). Accessed 23 Nov 2000.

[22] Radović AS, Usman S (2007). Voltage dependent pulse shape analysis of Geiger-Müller counter. Nucl. Technol..

[23] Model 44–7 Alpha-Beta-Gamma Detector. https://ludlums.com/products/all-products/product/model-44-7 (2019). Accessed 15 June 2019.

[24] 142A/B/C Preamplifiers, www.ortec-online.com/products/electronics/preamplifiers/142a-b-c (2020). Accessed 23 Nov 2000.

[25] Model 3125 0-2/0-5 kV Dual H.V. Power. http://www.nuclearphysicslab.com/npl/wp-content/uploads/Canberra_3125_Dual_HV_Power_Supply.pdf (2020). Accessed 22 July 2019.

[26] TBS2000 Digital Storage Oscilloscope. https://www.tek.com/oscilloscope/tbs2000-basic-oscilloscope (2020). Accessed 23 Nov 2000.

[27] Model 570 Spectroscopy Amplifier. https://www.ortec-online.com/products/electronics/amplifiers/570 (2020). Accessed 23 Nov 2000.

[28] Cardoso JM, Simões JB, Menezes T, Correia CMBA (1999). CdZnTe Spectra improvement through digital pulse amplitude correction using the linear sliding method. Nucl. Intstrm. Meth..

[29] Model 994 Dual Counter/Timer. https://www.ortec-online.com/products/electronics/counters-timers-rate-meter-and-multichannel-scaling-mcs/994. Accessed 23 Nov 2000 (2020)

